# A new nickel-based co-crystal complex electrocatalyst amplified by NiO dope Pt nanostructure hybrid; a highly sensitive approach for determination of cysteamine in the presence of serotonin

**DOI:** 10.1038/s41598-020-68663-2

**Published:** 2020-07-16

**Authors:** Hassan Karimi-Maleh, Fatemeh Karimi, Yasin Orooji, Ghobad Mansouri, Amir Razmjou, Aysenur Aygun, Fatih Sen

**Affiliations:** 1 School of Resources and Environment, University of Electronic Science and Technology of China, Xiyuan Ave, P.O. Box 611731, Chengdu, People’s Republic of China; 2 Department of Chemical Engineering, Laboratory of Nanotechnology, Quchan University of Technology, Quchan, Islamic Republic of Iran; 3 Department of Chemical Sciences, University of Johannesburg, Doornfontein Campus, P.O. Box 17011, Johannesburg, 2028 South Africa; 4 Nanostructure Based Biosensors Research Group, Ton Duc Thang University, Ho Chi Minh City, Vietnam; 5 Faculty of Applied Sciences, Ton Duc Thang University, Ho Chi Minh City, Vietnam; 6 Co-Innovation Center of Efficient Processing and Utilization of Forest Resources, Nanjing Forestry University, Nanjing, 210037 China; 7 College of Materials Science and Engineering, Nanjing Forestry University, Nanjing, 210037 People’s Republic of China; 8 Department of Chemistry, Payame Noor University, P.O. Box 19395-3697, Tehran, Iran; 9 Department of Biotechnology, Faculty of Biological Science and Technology, University of Isfahan, 81746-73441 Isfahan, Iran; 10 School of Chemical Science and Engineering, UNESCO Centre for Membrane Science and Technology, University of New South Wales, Sydney, 2052 Australia; 11 Department of Engineering, Macquarie University, Sydney, NSW 2109 Australia; 12 Sen Research Group, Biochemistry Department, Faculty of Arts and Science, Dumlupınar University, Evliya Çelebi Campus, 43100 Kütahya, Turkey

**Keywords:** Bioinorganic chemistry, Biochemistry, Chemistry, Materials science, Nanoscience and technology

## Abstract

A highly sensitive electrocatalytic sensor was designed and fabricated by the incorporation of NiO dope Pt nanostructure hybrid (NiO–Pt–H) as conductive mediator, bis (1,10 phenanthroline) (1,10-phenanthroline-5,6-dione) nickel(II) hexafluorophosphate (B,1,10,P,1,10, PDNiPF6), and electrocatalyst into carbon paste electrode (CPE) matrix for the determination of cysteamine. The NiO–Pt–H was synthesized by one-pot synthesis strategy and characterized by XRD, elemental mapping analysis (MAP), and FESEM methods. The characterization data, which confirmed good purity and spherical shape with a diameter of ⁓ 30.64 nm for the synthesized NiO–Pt–H. NiO–Pt–H/B,1,10, P,1,10, PDNiPF6/CPE, showed an excellent catalytic activity and was used as a powerful tool for the determination of cysteamine in the presence of serotonin. The NiO–Pt–H/B,1,10, P,1,10, PDNiPF6/CPE was able to solve the overlap problem of the two drug signals and was used for the determination of cysteamine and serotonin in concentration ranges of 0.003–200 µM and 0.5–260 µM with detection limits of 0.5 nM and 0.1 µM, using square wave voltammetric method, respectively. The NiO–Pt–H/B,1,10,P,1,10,PDNiPF6/CPE showed a high-performance ability for the determination of cysteamine and serotonin in the drug and pharmaceutical serum samples with the recovery data of 98.1–103.06%.

## Introduction

Cysteamine is a simple aminothiol with a variety of medicinal applications used to treat various diseases such as cystinosis and hypothyroidism^1^. Cysteamine is prescribed as a radiation-protective agent drug to treat radiation sickness^2^. Overdose of cysteamine brings about many side effects such as dizziness, rash, skin odor, headache, and tiredness. Therefore, the analysis of cysteamine should be monitored for administrated patients. On the other hand, taking cysteamine is effective in the serotonin neurotransmission level in body^3^. Also, the use of cysteamine in the treatment of patients can change tissue serotonin in duodenal ulceration^4^. Therefore, the simultaneous measurement of these two compounds is pivotal in the patients treated with cysteamine. Due to the important analysis of these compounds, many researchers focused on the fabrication of analytical sensors for their determination in biological and clinical samples^5–9^. Although there have been no reports on the simultaneous measurement of cysteamine and serotonin, various analytical methods such as chromatography, spectroscopy, and electrochemical sensors have been separately reported for each of these compounds^8,10–13^. Due to simple operations and low-cost analysis systems, electrochemical sensors have been widely used for the analysis of pharmaceutical and biological compounds^14–21^. Near over-potential oxidation of cysteamine and serotonin on the surface of a bare electrode is one of the most important problems in the simultaneous analysis of cysteamine and serotonin, using electrochemical methods^22^. Therefore, the application of electro-catalyst is necessary for resolving this issue in the simultaneous analysis^23–30^. EC^/^ electrochemical mechanism is a suitable strategy for the determination of two analytes with near over-potential in electrochemical analysis systems^31–37^. The interaction of one analyte with a suitable electrocatalyst and remaining of another analyte at its potential is a very interesting strategy based on electrocatalytic systems in the simultaneous analysis^37–41^. Selecting a suitable catalyst with high selectivity is one of the most important steps in the design of electrocatalytic sensors^42–46^. According to previous reports, inorganic complexes, especially complexes with nickel central atom can be used as a suitable electrocatalyst in the analysis of important pharmaceutical and biological constituents^47–52^. Although nanomaterials such as carbon nanotubes and graphene with high-conductivity have been proposed for the preparation of electrochemical sensors, their high cost, hard synthesis methods, and high capacitive charging current are some of the most important criteria for their use in electrochemical sensors^53–61^. Accordingly, the use of metal oxides has been used as a suitable alternative to this category of materials^62–66^. Recent research suggests that modifying metal oxides with metal nanoparticles such as platinum could increase the electrical conductivity of the sensors and create the right conditions for the design of high-sensitivity sensors^67^.

Therefore, in this study, we fabricated NiO–Pt–H/B,1,10,P,1,10,PDNiPF6/CPE as a first and highly sensitive electroanalytical sensor for simultaneous determination of cysteamine and serotonin. The NiO–Pt–H/B,1,10,P,1,10,PDNiPF6/CPE was successfully used for the determination of cysteamine and serotonin in real samples.

## Experiment

### Instruments and materials

Electrochemical signals were recorded by Potentiostat/Galvanostat Electrochemical Instrument PGSTAT-302N (Netherlands). The Field Emission SEM machine model Mira 3-XMU was used for morphological and EDS analysis of NiO dope Pt nanostructure hybrid. The structural analysis of NiO dope Pt nanostructure hybrid was characterized by XRD machine model X’Pert Pro. The Ag/AgCl/KClsat and electrochemical cell were purchased from Azar Electrode Company.

Cysteamine hydrochloride and serotonin hydrochloride were purchased from Sigma-Aldrich Company. The 0.01 M stock solution of cysteamine hydrochloride and serotonin hydrochloride was prepared by dissolving 0.113 g and 0.212 g compounds in 100 mL phosphate buffer solution (PBS) pH 7.0.

Nickel nitrate hexahydrate, sodium hydroxide, and platinum (II) chloride were purchased from Merck Company and used for one-pot synthesis of NiO dope Pt nanostructure hybrid. Phosphoric acid was purchased from Acros Company and used for the preparation of PBS.

### Synthesis of NiO dope Pt nanostructure hybrid

The 100-mL solution containing 16 mg platinum (II) chloride and 0.5 M nickel nitrate hexahydrate were stirred for 30 min at room temperature. The 100-mL sodium hydroxide 1.0 M was added to the previous solution over a 1.5 h period. The green precipitated sample was filtered and then dried at 15 h at 100 °C. The green powder was calcined at 400° C in a furnace for 3 h.

### Preparation of NiO–Pt–H/B,1,10,P,1,10,PDNiPF6/CPE

The NiO–Pt–H/B,1,10,P,1,10,PDNiPF6/CPE was designed and made by using slurry composed of 0.95 gr of graphite powder 0.04 g + NiO–Pt–H and 0.05 g of B,1,10,P,1,10,PDNiPF6 using 13 drops of paraffin oil as binders into mortar and pestle in the presence of 10 mL ethanol. After evaporation of the ethanol solvent, the mixture was hand-mixed for 90 min to obtain a homogeneous paste.

### Preparation of real sample

Cysteamine capsules (150 mg) were purchased from a local pharmacy and after opening 10 capsules, the powder was dissolved into a 100-mL solution containing ethanol/PBS 1:1 and stirred for 1 h. Dilution was performed using phosphate buffer solution.

### Recommend procedure

Electrochemical behavior of cysteamine was investigated by recording cyclic voltammograms of 500 µM cysteamine on the surface of NiO–Pt–H/B,1,10,P,1,10,PDNiPF6/CPE, B,1,10,P,1,10,PDNiPF6/CPE, NiO–Pt–H/CPE and CPE at pH 7.0 and scan rate 10 mV/s. Then, the recorded cyclic voltammograms were compared together for the investigation catalytic effect of the fabricated sensors.

Interference study was investigated by recording square wave voltammogram (SWV) of 20.0 µM cysteamine on the surface of NiO–Pt–H/B,1,10,P,1,10,PDNiPF6/CPE. The 20.0 µM in the electrochemical cell containing 10 mL buffer solution was equal to 2.2 × 10^–6^ g of cysteamine in solution. In the next step and after the addition of interference with a maximum acceptable value of 1,000 fold (w/w) into an electrochemical cell, the signal of the solution containing cysteamine and interference was recorded. The 5% error in current was acceptable and showed that interference did not have a significant effect on the analysis signal.

Stability of NiO–Pt–H/B,1,10,P,1,10,PDNiPF6/CPE as an electroanalytical sensor for determination of cysteamine was investigated by recording SWV of 100.0 µM cysteamine over a course of 60 days on the surface of the fabricated sensor. The drug signal was recorded every ten days and the current obtained was compared with the initial current of the drug.

## Results and discussion

### Characterization of NiO–Pt–H

**Figure 1:**
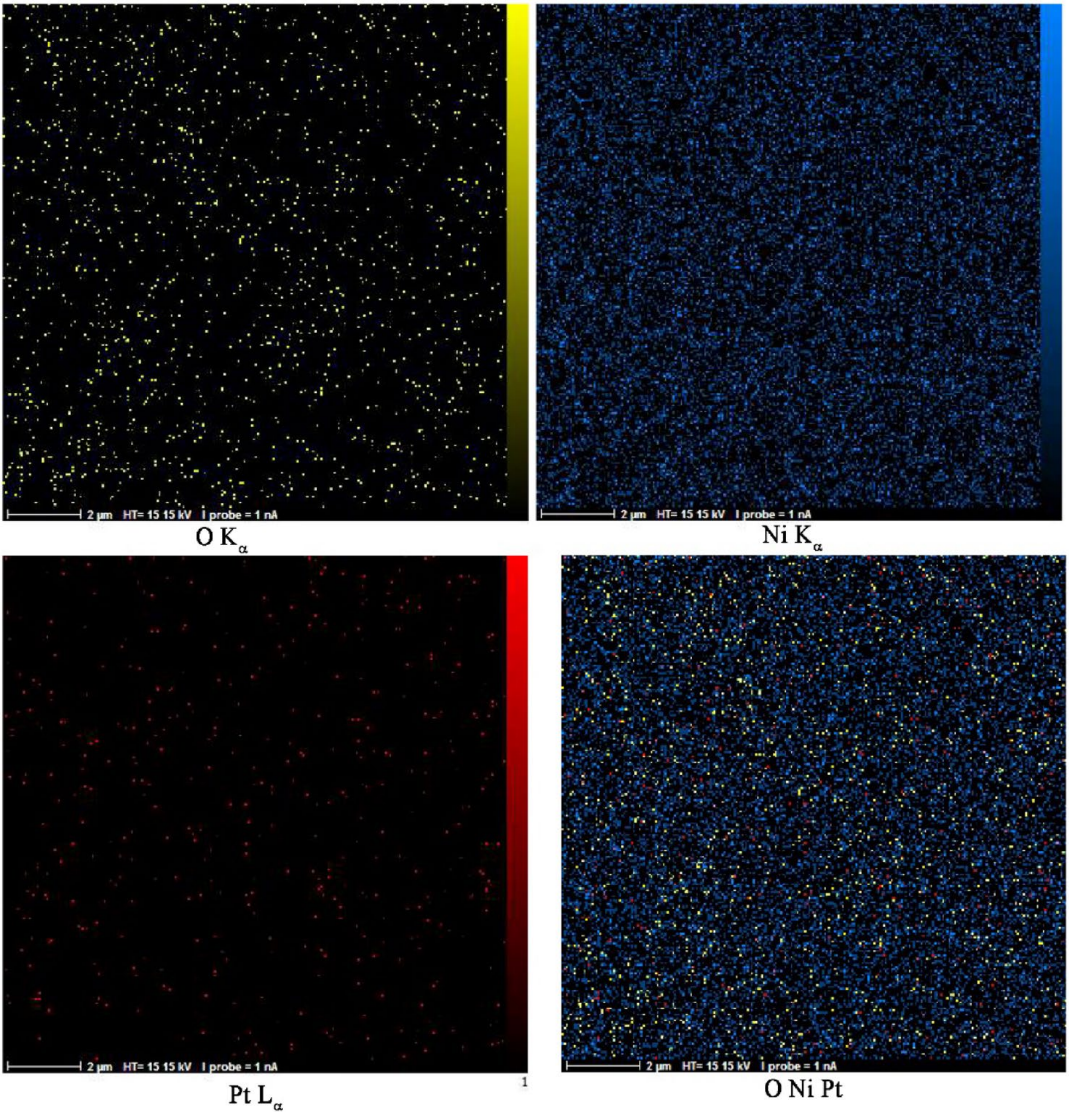
MAP analysis data for synthesized NiO dope Pt nanostructure hybrid.

**Figure 2:**
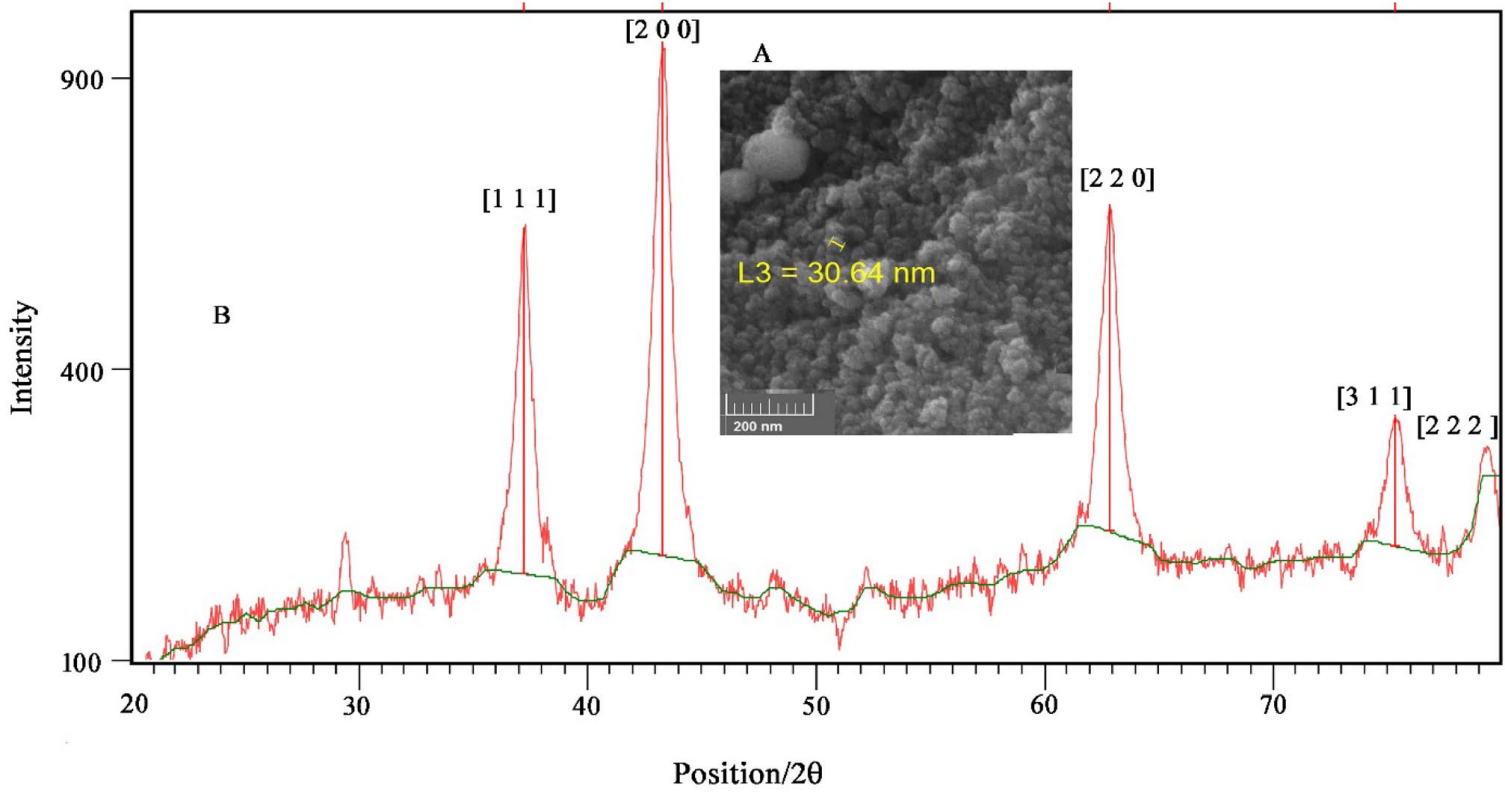
**(A)** FESEM image and **(B)** XRD pattern of NiO dope Pt nanostructure hybrid.

The purity and particle distribution of NiO–Pt–H were characterized by MAP analysis. The results are presented in Fig. 1, showing good distribution and purity of the synthesized NiO–Pt–H. In addition, the FESEM figure showed a spherical shape for synthesized NiO–Pt–H with good distribution in nanoparticle sizes (Fig. 2A). Furthermore, the XRD pattern of NiO–Pt–H showed planes with miller indexes of (1 1 1), (2 0 0), (2 2 0), (3 1 1), and (2 2 2) relative to NiO particle with a code No. 04-0835. On the other hand, due to the low concentration of Pt in a synthesized nano-hybrid, the Pt planes could not be detected in XRD pattern (Fig. 2B).

### Electrocatalytic determination of cysteamine using NiO–Pt–H/B,1,10,P,1,10,PDNiPF6/CPE

**Figure 3:**
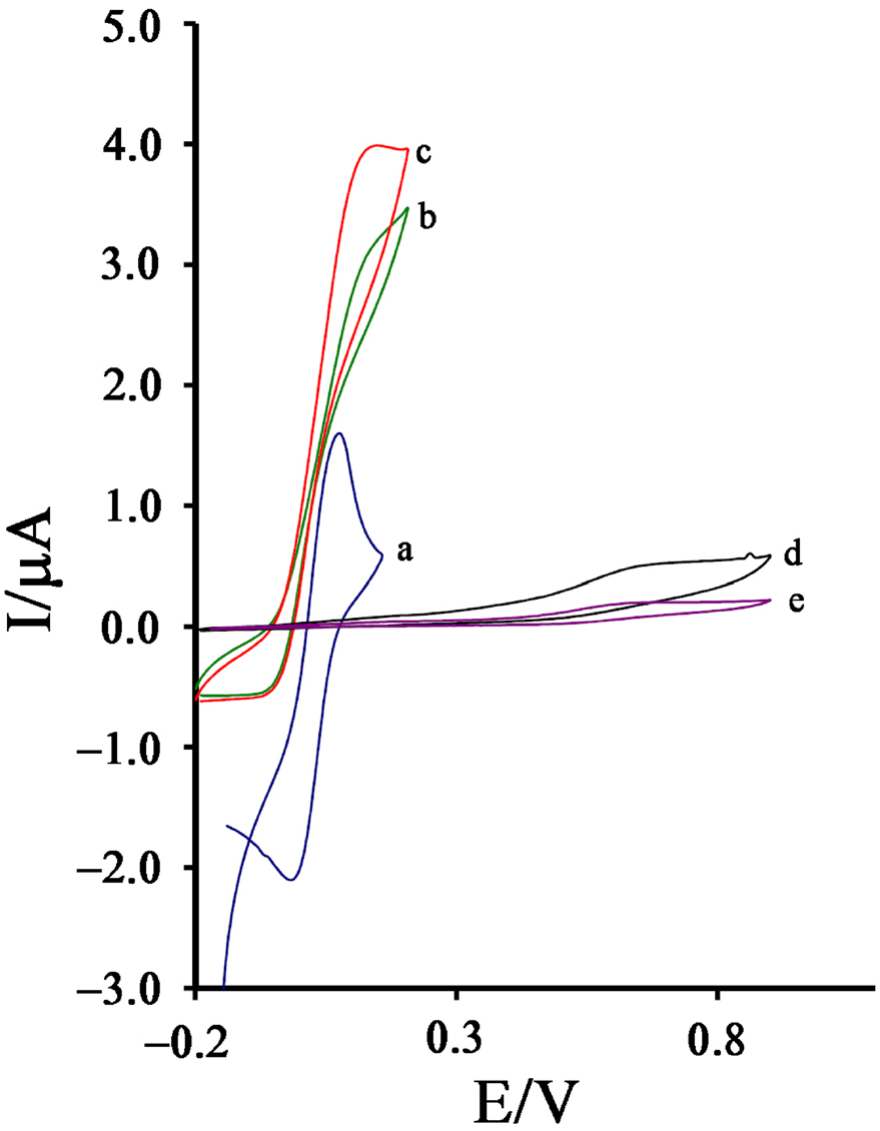
**(a)** Cyclic voltammogram of NiO–Pt–H/B,1,10,P,1,10,PDNiPF6/CPE in the PBS (pH 7.0). **(b)** Cyclic voltammogram of B,1,10,P,1,10,PDNiPF6/CPE in the presence of 500 µM cysteamine. **(c)** Cyclic voltammogram of NiO–Pt–H/B,1,10,P,1,10,PDNiPF6/CPE in the presence of 500 µM cysteamine. **(d)** Cyclic voltammogram of NiO–Pt–H/CPE in the presence of 500 µM cysteamine and **(e)** cyclic voltammogram of CPE in the presence of 500 µM cysteamine (condition; pH 7.0, scan rate 10 mV/s).

**Scheme 1:**
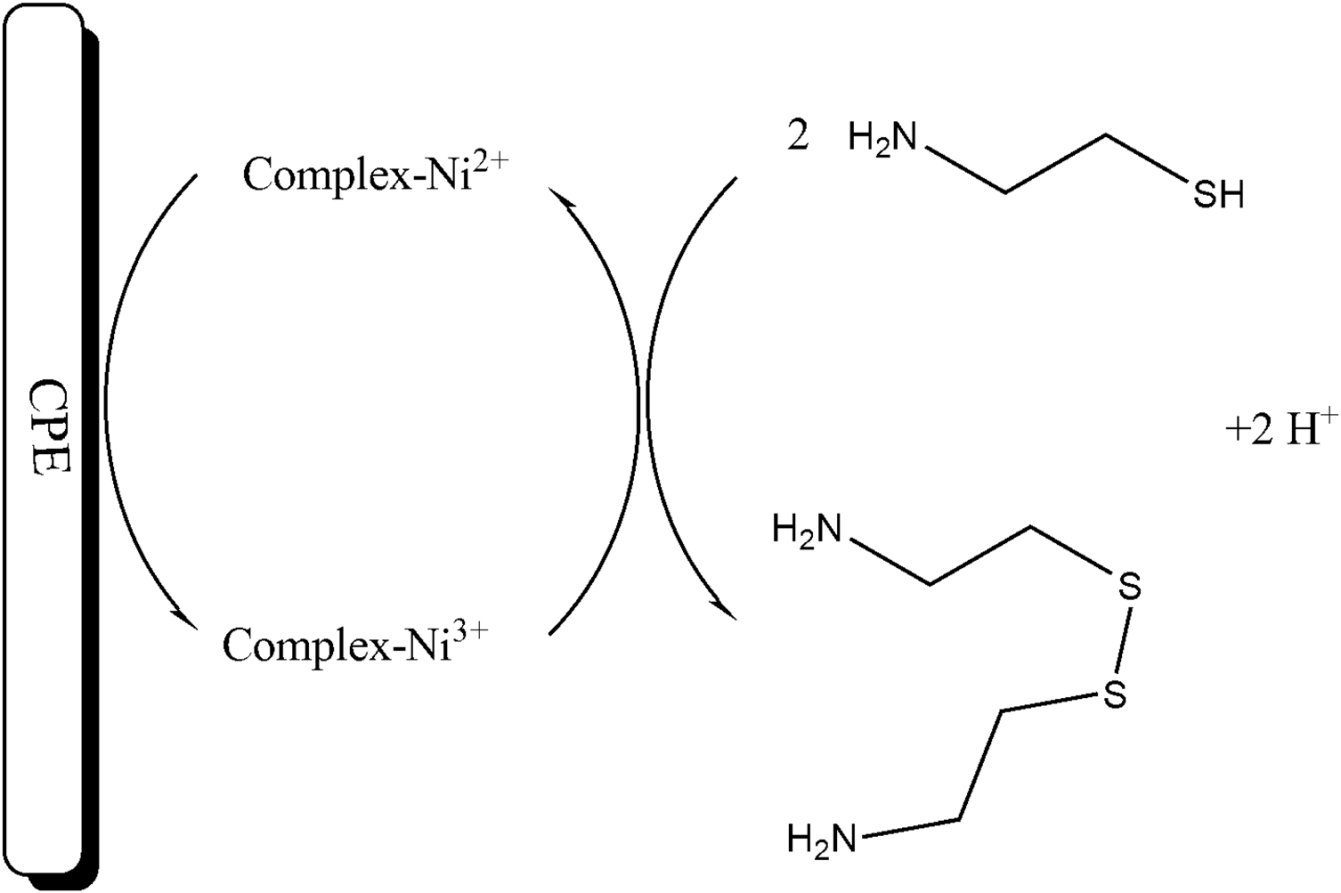
Electrocatalytic mechanism for determination of cysteamine on the surface of NiO–Pt–H/B,1,10,P,1,10,PDNiPF6/CPE.

The cyclic voltammograms NiO–Pt–H/B,1,10,P,1,10,PDNiPF6/CPE (Fig. 3, curve a) was recorded in the phosphate buffer solution (pH 7.0). Recording voltammogram showed a redox signal with quasi behavior (ΔE = 73 mV) relative to Ni^2+^/Ni^3+^ in the absence of cysteamine. After the addition of 500 µM cysteamine on the surface of NiO–Pt–H/B,1,10,P,1,10,PDNiPF6/CPE, the oxidation current of B,1,10,P,1,10,PDNiPF6 increased and the reduction signal of mediator was removed (E_Oxidation_ ~ 120 mV). This phenomenon exhibits a kind of electrocatalytic behavior between the intermediate (B,1,10,P,1,10,PDNiPF6 in this case) and the cysteamine (see Scheme 1). On the other hand, on the surface of B,1,10,P,1,10,PDNiPF6/CPE (Fig. 3, curve c), the same electrocatalytic behavior with a weaker signal was observed, which can be attributed to the role of nanoparticles on the electrode surface. In the same solution and on the surface of CPE (Fig. 3, curve e), a low oxidation signal at potential ~ 620 mV relative to electrooxidation of cysteamine can be observed.

After modification of CPE with NiO–Pt–H and on the surface of NiO–Pt–H/CPE, the oxidation signal of cysteamine was increased, but a small decrease was observed in oxidation potential of cysteamine (Fig. 3, curve d). Comparison of curve “c” with curve “e” properly indicates that measurement of cysteamine at a potential of about 500 mV is less positive than its actual value at unmodified electrode with greater sensitivity which can be possible on the surface of NiO–Pt–H/B,1,10,P,1,10,PDNiPF6/CPE.

**Figure 4:**
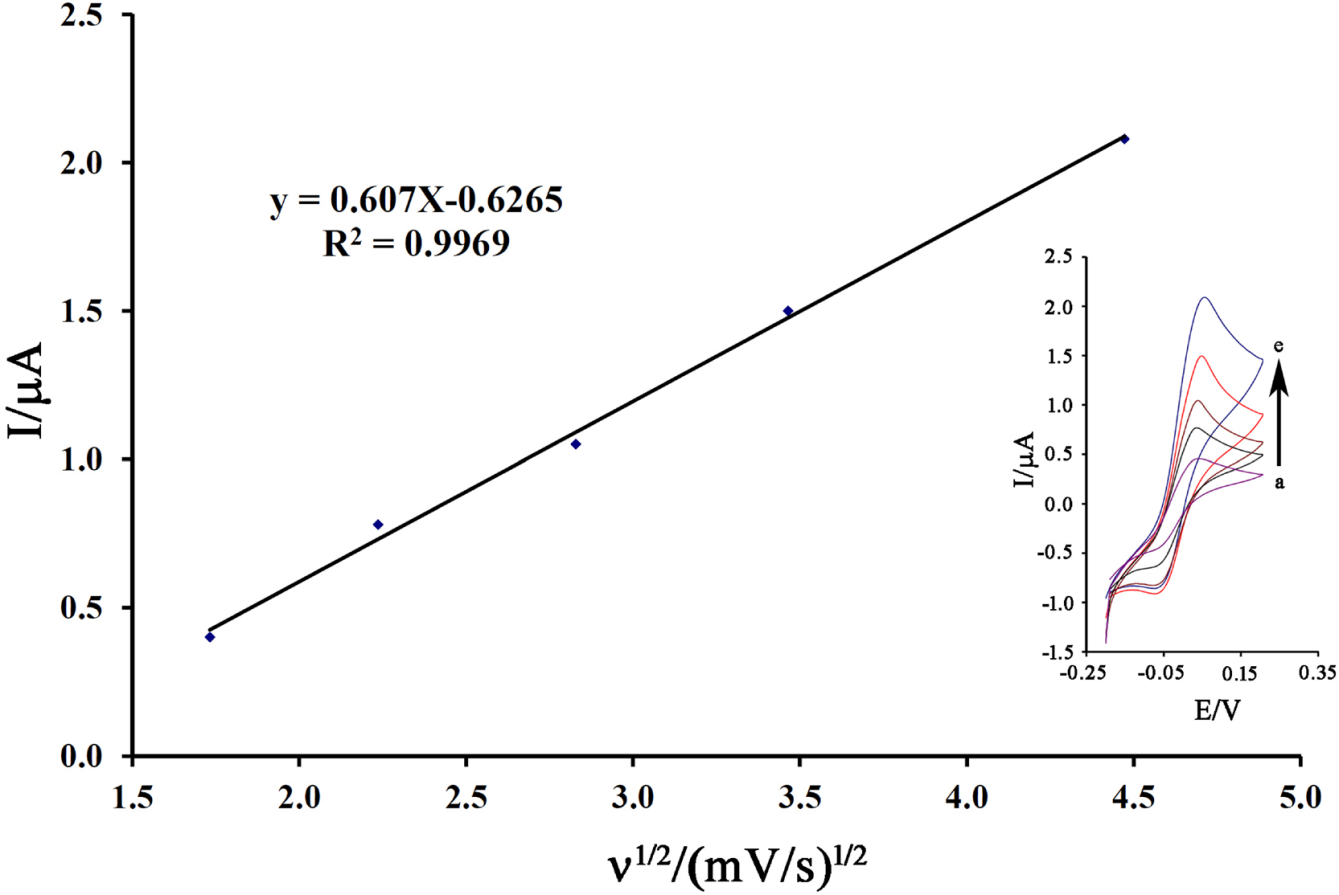
The plot of I vs. ν^1/2^ for electro-oxidation 200 µM cysteamine on the surface of NiO–Pt–H/B,1,10,P,1,10,PDNiPF6/CPE in the scans **(a)** 3; **(b)** 5; **(c)** 8; **(d)** 12 and **(e)** 20 mV/s.

The cyclic voltammograms 200 µM cysteamine on the surface of NiO–Pt–H/B,1,10,P,1,10,PDNiPF6/CPE in the scan range of 3.0–20.0 mV/s was recorded and the results are presented in Fig. 4 inset. According to equation I_pa_ = 0.607 ν^1/2^ − 0.6265 (R^2^ = 0.9969), a linear relation was observed between the oxidation signal of cysteamine and ν^1/2^ on the surface of NiO–Pt–H/B,1,10,P,1,10,PDNiPF6/CPE (Fig. 4). This linear relation confirms a diffusion process^68–70^ for electro-oxidation of cysteamine on the surface of NiO–Pt–H/B,1,10,P,1,10,PDNiPF6/CPE.

**Figure 5:**
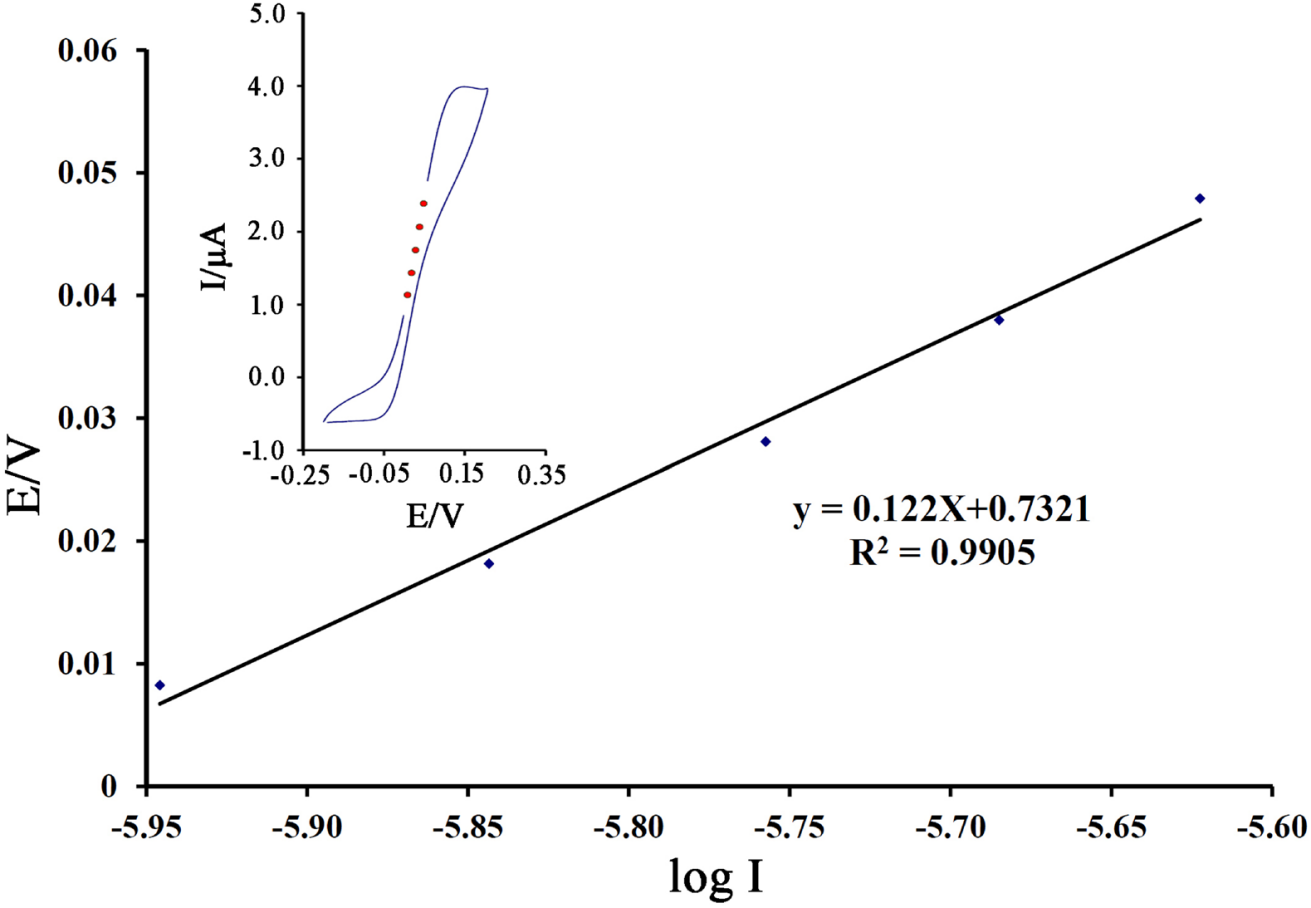
Tafel plot for NiO–Pt–H/B,1,10,P,1,10,PDNiPF6/CPE in (pH 7.0) with a scan rate of 10 mV/s in the presence of 500 µM cysteamine.

The value electron transfer coefficient (α) as a kinetic parameter, containing useful information about the rate-determining step, was calculated by the Tafel plot (Fig. 5). The slope of the Tafel plot relative electrooxidation 600 µM cysteamine on the surface of NiO–Pt–H/B,1,10,P,1,10,PDNiPF6/CPE was equal to 2.3RT/n(1 − α)F, which came up to 0.122 V decade^−1^ for scan rates of 10 mV s^−1^. The value of α for cysteamine was determined as ~ 0.52.

**Figure 6:**
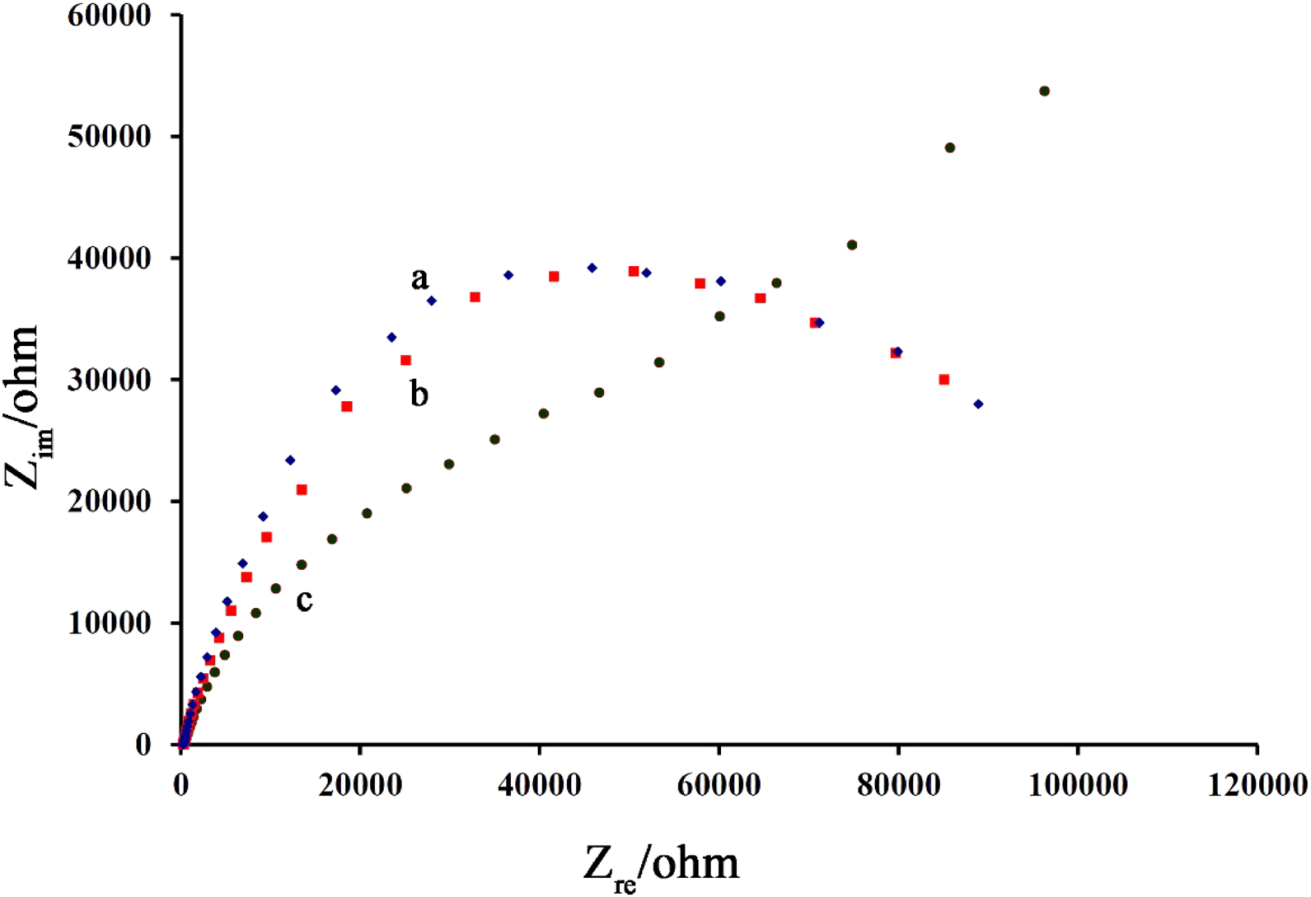
Nyquist diagrams of NiO–Pt–H/B,1,10,P,1,10,PDNiPF6/CPE in the absence **(a)** and in the presence **(b)** of 1.0 mM serotonin and **(c)** with 1.0 mM cysteamine.

The Electrochemical impedance spectroscopy (EIS) technique was used to confirm the electro-catalytic process between B,1,10,P,1,10,PDNiPF6 and cysteamine on the surface of NiO–Pt–H/B,1,10,P,1,10,PDNiPF6/CPE (Fig. 6). The Nyquist diagrams of NiO–Pt–H/B,1,10,P,1,10,PDNiPF6/CPE in the absence (curve a) and presence of 1.0 mM cysteamine (curve b) and 1.0 mM serotonin (curve c) are presented in Fig. 6, respectively. As can be seen, the diameter of the semicircle (relative to charge transfer resistance) relative to NiO–Pt–H/B,1,10,P,1,10,PDNiPF6/CPE in the absence and presence of 1.0 mM serotonin are very similar, confirming that no electrocatalytic reaction took place between serotonin and mediator on the surface of NiO–Pt–H/B,1,10,P,1,10,PDNiPF6/CPE. After the addition of 1.0 mM cysteamine (curve b), the diameter of the semicircle was decreased on the surface of NiO–Pt–H/B,1,10,P,1,10,PDNiPF6/CPE. This point is relative to the electrocatalytic reaction between mediator and cysteamine, increasing in oxidation signal of the mediator and decreasing in charge transfer resistance of electrode surface.

### Chronoamperometric investigation

**Figure 7:**
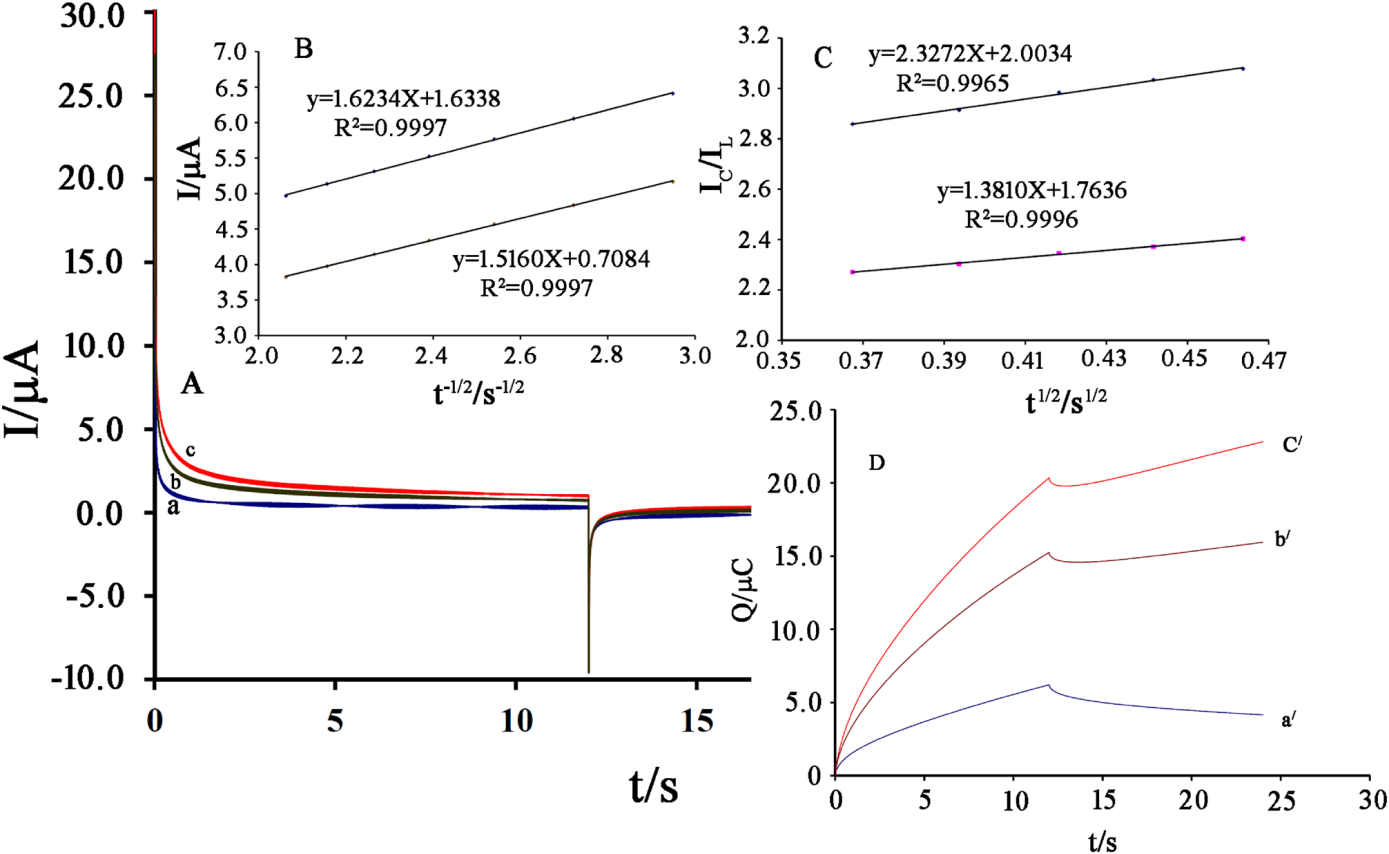
**(A)** Chronoamperograms obtained at NiO–Pt–H/B,1,10,P,1,10,PDNiPF6/CPE **(a)** in the absence, **(b)** in the presence of 50.0 µM and **(c)** in the presence of 100.0 cysteamine at pH 7.0. **(B)** Cottrell’s plot for cysteamine oxidation data on the surface of NiO–Pt–H/B,1,10,P,1,10,PDNiPF6/CPE. **(C)** Dependence of I_c_/I_L_ on the t^1/2^ derived from the chronoamperogram data. **(D)** The charge–time curves derived from the chronoamperogram data.

The value of diffusion coefficient (D) and catalytic rate constant, kh were determined using chronoamperogram recording of NiO–Pt–H/B,1,10,P,1,10,PDNiPF6/CPE in the absence of (Fig. 7A, curve a) and in the presence of 50 µM (Fig. 7A, curve b) and 100 µM (Fig. 7A, curve c) cysteamine, using applied potential − 0.1 and 0.2 mV. The value of D was determined by recording Cottrell plots relative to chronoamperogram which was recorded in the presence of cysteamine (Fig. 7B). The slopes and Cottrell equation (Eq. 1):

1 $$I=\frac{nFA{D}^{1/2}C}{{\pi }^{1/2}}{t}^{-1/2} $$

The mean value of D for cysteamine was determined as ~ 2.45 × 10^–5^ cm^2^/s.

The catalytic rate constant between B,1,10,P,1,10,PDNiPF6 and cysteamine can be determined according to the method of Galus equation (Eq. 2):

2 $$\frac{{I}_{C}}{{I}_{L}}={\gamma }^{{1/2}}{\pi }^{{1/2}}={\pi }^{{1/2}}{\left({k}_{h}Ct\right)}^{{1/2}} $$

Based on the slope reported from the I_C_/I_L_ (I_C_ is catalytic current in the presence of cysteamine and I_L_ oxidation current in the absence of cysteamine) vs. t^1/2^ and Eq. (2), the value of k_h_ can be determined 1.4732 × 10^4^ mol^−1^ L s^−1^ (Fig. 7C).

Chronocoulometry technique (double potential step) was also used for the examination of electrode processes at NiO–Pt–H/B,1,10,P,1,10,PDNiPF6/CPE (Fig. 7D). Forward and backward charge on the NiO–Pt–H/B,1,10,P,1,10,PDNiPF6/CPE in a blank buffer solution showed very symmetrical chronocoulograms. This recorded signal confirms an equal charge consumed for redox reaction of the Ni^2+^/Ni^3+^ system in NiO–Pt–H/B,1,10,P,1,10,PDNiPF6/CPE. However, after the addition of cysteamine, the value of oxidation charge value in chronocoulometric investigation was increased and the backward charge value in chronocoulometric investigation was decreased. These changes properly illustrate the electrocatalytic process.

### Simultaneous determination of cysteamine and serotonin

The square wave voltammograms (SWV) of cysteamine and serotonin were recorded separately on the surface of NiO–Pt–H/B,1,10,P,1,10,PDNiPF6/CPE. The results showed a linear relation between oxidation current of cysteamine and its concentration in the range of 0.003–200 µM with equation I_pa_ = 0.0385 C_cysteamine_ + 0.9602 (R^2^ = 0.9978) and a linear range between 0.5 and 260 µM with equation I_pa_ = 0.0238 C_Serotonin_ + 0.4139 (R^2^ = 0.9969) for the determination of serotonin on the surface of NiO–Pt–H/B,1,10,P,1,10,PDNiPF6/CPE. The detection limit (LOD) was 0.5 nM cysteamine and 0.1 µM serotonin, according to the definition of LOD = 3sb/m.

**Figure 8:**
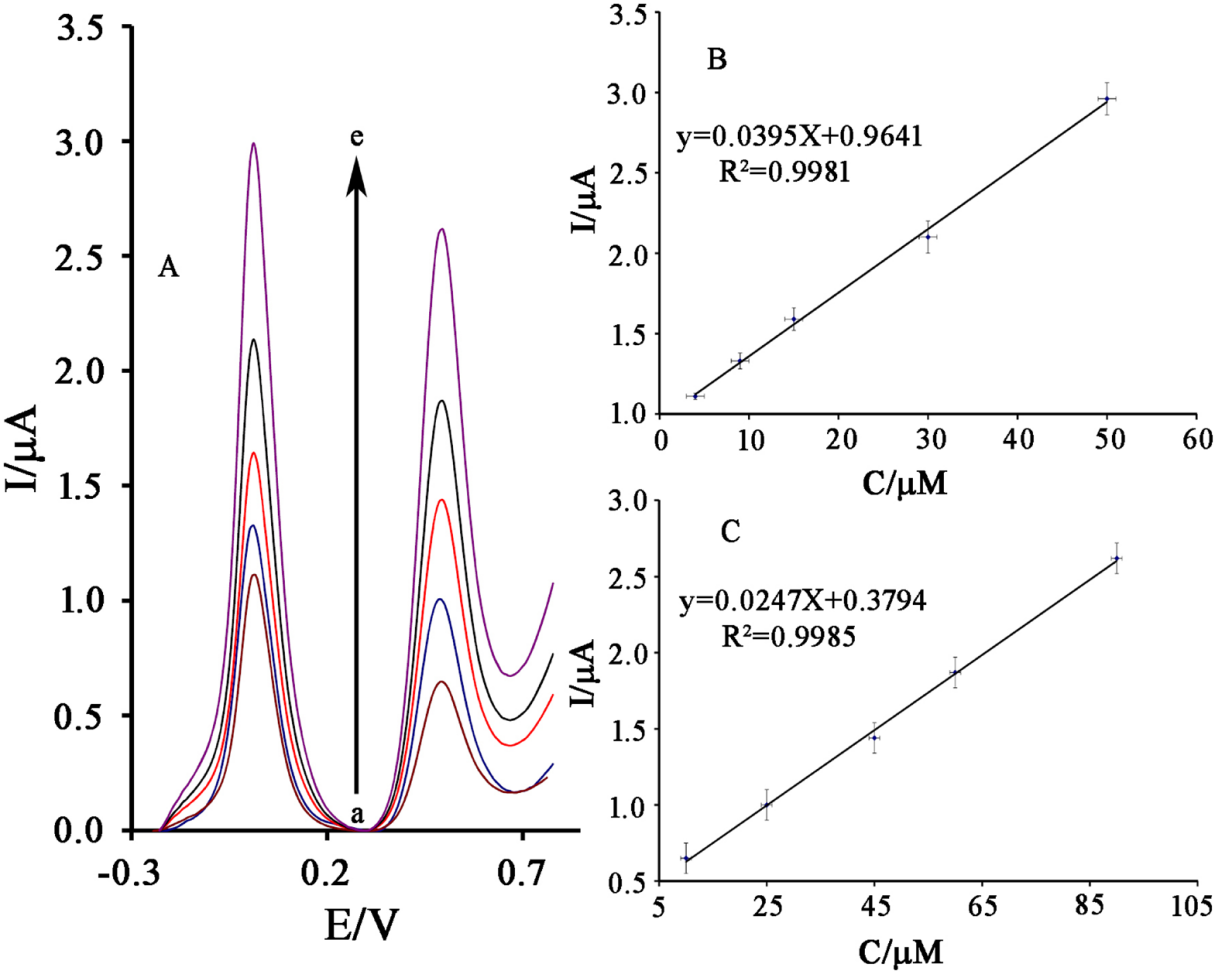
**(A)** SWVs of NiO–Pt–H/B,1,10,P,1,10,PDNiPF6/CPE containing different concentrations of cysteamine–serotonin in µM (from inner to outer): 4.0 + 10.0; 9.0 + 25.0; 15.0 + 45.0; 30.0 + 60.0 and 50.0 + 90.0, respectively. Insets: Plots of I_p_ vs. **(B)** cysteamine and **(C)** serotonin concentrations.

**Figure 9:**
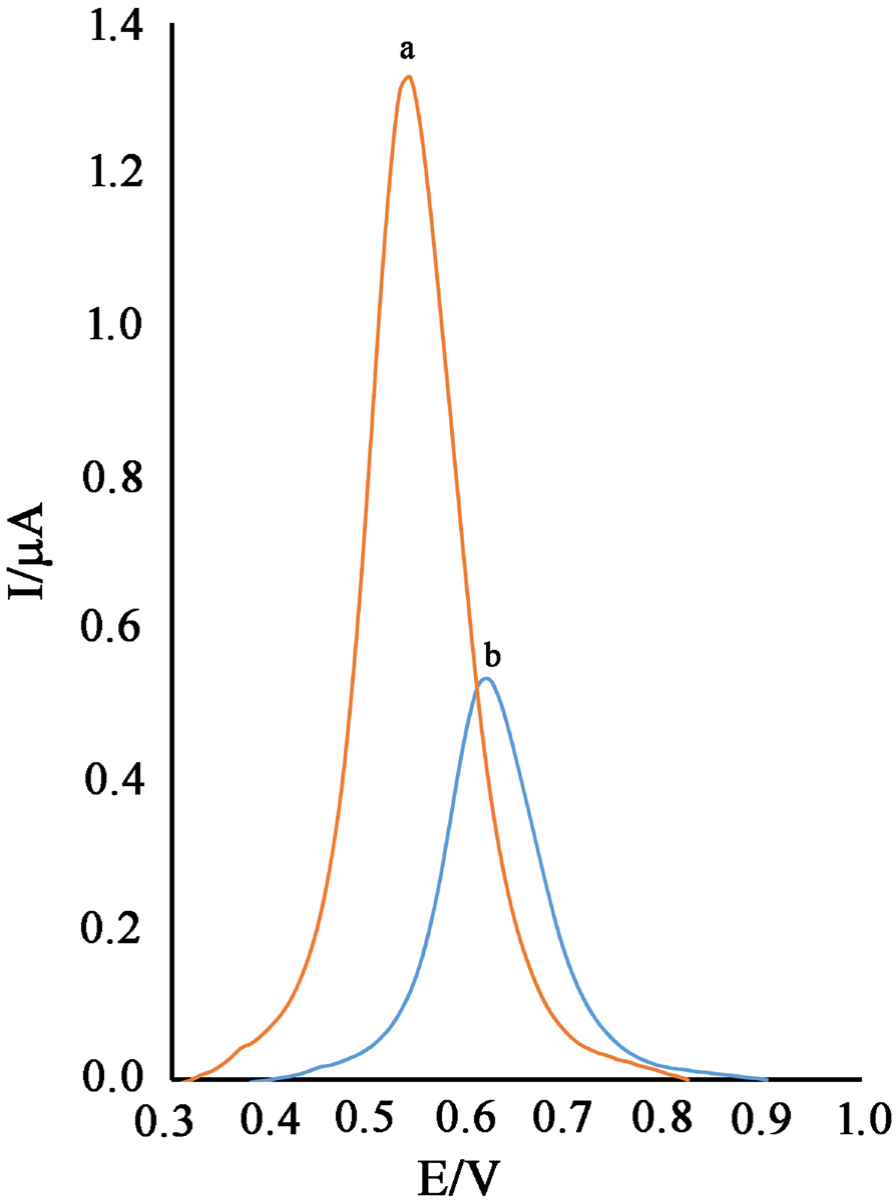
SWVs of carbon paste electrode in the solution containing **(a)** 500 µM serotonin and **(b)** 500 µM cysteamine.

On the other hand, the square wave voltammograms of NiO–Pt–H/B,1,10,P,1,10,PDNiPF6/CPE in the solution containing different concentrations of cysteamine and serotonin was recorded and the obtained signals were presented in Fig. 8A. As can be seen, we detected two separated oxidation signals relative to cysteamine and serotonin at potentials of 10 mV and 495 mV with ΔE = 485 mV that is very interesting for the simultaneous determination of the two compounds using NiO–Pt–H/B,1,10,P,1,10,PDNiPF6/CPE. On the other hand, the oxidation potential of cysteamine and serotonin is very close to each other on the surface of the carbon paste electrode (Fig. 9). The linear relation between oxidation signal of cysteamine and serotonin and their concentration in this investigation are presented in Fig. 8B, C. Slopes of 0.0395 µA/µM and 0.0247 µA/µM for cysteamine and serotonin were obtained, respectively. These sensitivities are very similar to the sensitivity recorded for cysteamine and serotonin in linear dynamic range investigation, confirming that the determination of cysteamine and serotonin can be done successfully without any interference in the mixed samples.

### Stability and reproducibility

**Figure 10:**
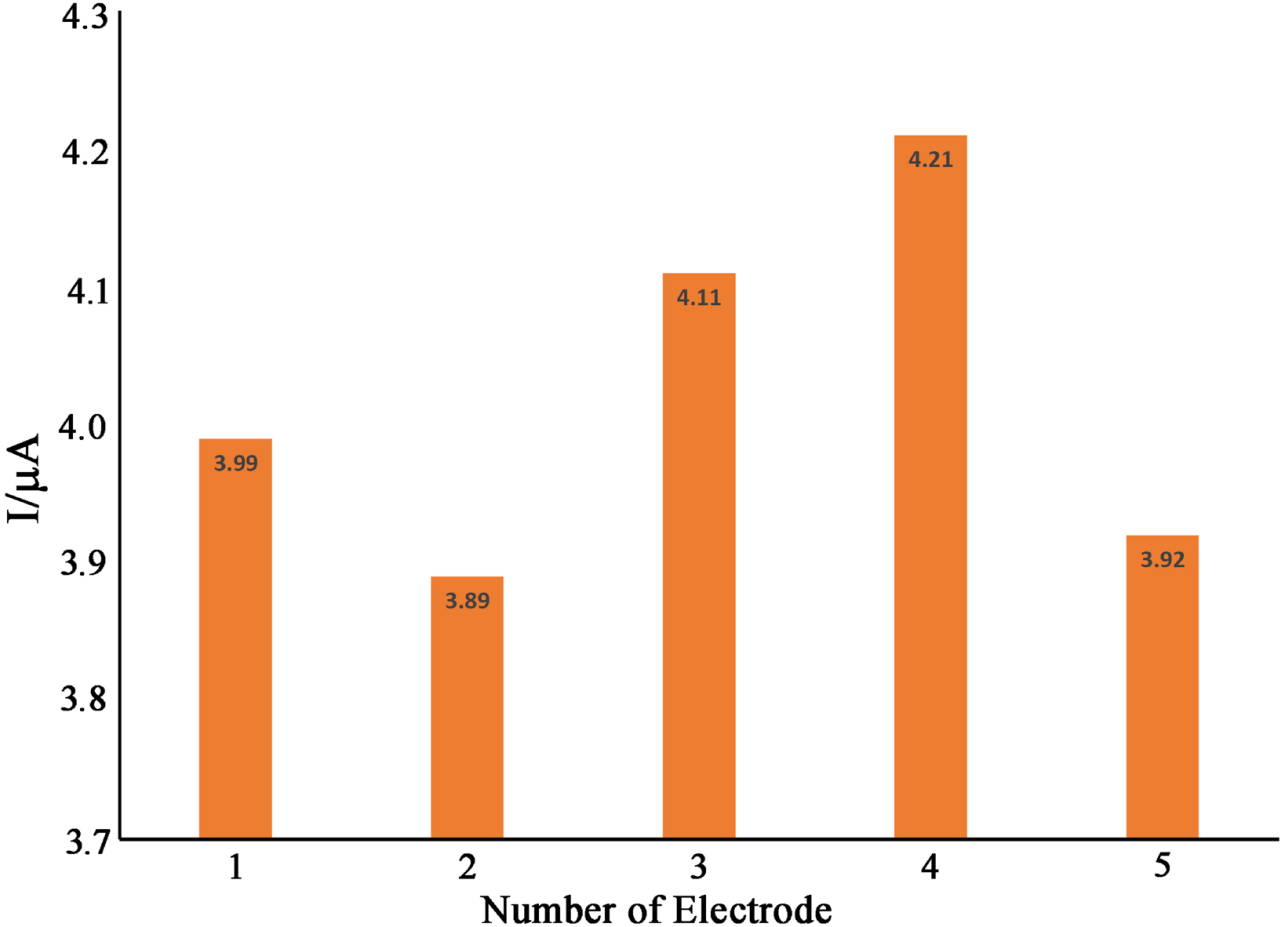
Recorded current for electrooxidation of 500 µM cysteamine on the surface of five different NiO–Pt–H/B,1,10,P,1,10,PDNiPF6/CPEs prepared in the same condition.

The reproducibility and stability of NiO–Pt–H/B,1,10,P,1,10,PDNiPF6/CPE were investigated by recording SWV of 100.0 µM cysteamine at pH 7.0. We detected a relative standard deviation of (RSD%) 2.1% for five successive recorded signals that confirmed good repeatability for NiO–Pt–H/B,1,10,P,1,10,PDNiPF6/CPE as an electroanalytical sensor. Moreover, the stability of the NiO–Pt–H/B,1,10,P,1,10,PDNiPF6/CPE was examined by the storage of the sensor in the lab. Then, NiO–Pt–H/B,1,10,P,1,10,PDNiPF6/CPE was used for the determination of 100.0 µM cysteamine using SWV. The recorded signal showed 93.2% of its initial response relative to 100.0 µM cysteamine after 60 days, using NiO–Pt–H/B,1,10,P,1,10,PDNiPF6/CPE, indicating good stability for the suggested sensor. To study the reproducibility of NiO–Pt–H/B,1,10,P,1,10,PDNiPF6/CPE in determination of 500 µM cysteamine, five modified electrodes were prepared in the same condition and the oxidation signal of cysteamine was recorded on the surface of NiO–Pt–H/B,1,10,P,1,10,PDNiPF6/CPE with a scan rate of 10 mV/s. A relative standard deviation of (RSD%) 3.33% was detected for the determination of cysteamine on the surface of these electrodes, confirming good reproducibility for the fabrication of NiO–Pt–H/B,1,10,P,1,10,PDNiPF6/CPE (Fig. 10).

### Real sample and interference study

The selectivity of NiO–Pt–H/B,1,10,P,1,10,PDNiPF6/CPE as an electroanalytical sensor was checked in the solution containing 20.0 µM cysteamine with an acceptable error of 5% in oxidation current. The results are presented in Table 1 and the data obtained exhibit interesting selection of NiO–Pt–H/B,1,10,P,1,10,PDNiPF6/CPE as an electroanalytical sensor for the determination of cysteamine.

**Table 1:** The interference study results in the presence of 20.0 µM cysteamine.

Species	Tolerant limits (W_interference_/W_cysteamine_)
Cl^-^, K^+^, Li^+^, F^-^, Mg^2+^	950
Fructose and glucose, ascorbic acid (after addition of 1.0 mM ascorbate oxidase)	750
Methionine, Dopamine, Valine, Uric acid, vitamin B_2_	300

In the final step, we check the ability of NiO–Pt–H/B,1,10,P,1,10,PDNiPF6/CPE as a powerful electroanalytical sensor for determination of cysteamine and serotonin in capsule and pharmaceutical serum samples. The recovery data between 98.5–103.06% for analysis of cysteamine and 98.1–101.68% for analysis of serotonin were observed on the surface of NiO–Pt–H/B,1,10,P,1,10,PDNiPF6/CPE (Table 2), confirming the powerful ability of NiO–Pt–H/B,1,10,P,1,10,PDNiPF6/CPE as a novel and powerful analytical sensor.

**Table 2:** Determination of cysteamine and serotonin in real samples (n = 5).

Samples	Cysteamine standard solution added (µM)	Serotonin standard solution added (µM)	Founded of cysteamine (µM)	Founded of serotonin (µM)	Recovery value % for cysteamine	Recovery value % for serotonin
Cysteamine capsule	–	–	–	4.95 ± 0.45	–	–
	5.00	–	9.83 ± 0.67	–	98.79	–
Pharmaceutical serum	**–**	**–**	< LOD	< LOD	**–**	**–**
	10.00	10.00	9.85 ± 0.39	9.81 ± 0.52	98.5	98.1
	15.00	45.00	15.46 ± 0.59	45.76 ± 0.93	103.06	101.68

## Conclusion

In this study, a novel and highly sensitive electroanalytical sensor was fabricated for the determination of cysteamine by modification of CPE with NiO–Pt–H and B,1,10,P,1,10,PDNiPF6 as two mediators. The NiO–Pt–H was synthesized by an one-pot procedure resulting in a spherical shape with diameter of 30.64 nm. The NiO–Pt–H/B,1,10,P,1,10,PDNiPF6/CPE was used as the first electrochemical sensor for the simultaneous analysis of cysteamine and serotonin with detection limits of 0.5 nM and 0.1 µM, respectively. In addition, NiO–Pt–H and B,1,10,P,1,10,PDNiPF6 showed a powerful ability for the determination of cysteamine and serotonin in the drug and pharmaceutical serum samples with recovery data of 98.1–103.06%.
